# Expression and function of microRNA-9 in the mid-hindbrain area of embryonic chick

**DOI:** 10.1186/s12861-017-0159-8

**Published:** 2018-02-22

**Authors:** A. Alwin Prem Anand, Carola Huber, John Asnet Mary, Nancy Gallus, Christoph Leucht, Ruth Klafke, Bernhard Hirt, Andrea Wizenmann

**Affiliations:** 1 Institute of Clinical Anatomy and Cell Analysis, University of Tuebingen, Oesterbergstrasse 3, D-72074 Tuebingen, Germany; 2 Department of Zoology, Fatima College, Madurai, Tamilnadu 625018 India; 3 Institute of Developmental Genetics, Helmholtz Zentrum München, Deutsches Forschungszentrum für Gesundheit und Umwelt (GmbH), Ingolstädter Landstr. 1, 85764 Neuherberg, Germany; 4 Robert-Bosch-Krankenhaus, Auerbachstraße 110, 70376 Stuttgart, Germany; 5 Department of Neurobiology, McKnight Brain Institute, University of Alabama at Birmingham, Birmingham, AL 35294 USA

**Keywords:** microRNA, Mid-hindbrain area, Chick, miR-9, Brain development

## Abstract

**Background:**

MiR-9 is a small non-coding RNA that is highly conserved between species and primarily expressed in the central nervous system (CNS). It is known to influence proliferation and neuronal differentiation in the brain and spinal cord of different vertebrates. Different studies have pointed to regional and species-specific differences in the response of neural progenitors to miR-9.

**Methods:**

*In ovo* and *ex ovo* electroporation was used to overexpress or reduce miR-9 followed by mRNA in situ hybridisation and immunofluorescent stainings to evaluate miR- expression and the effect of changed miR-9 expression.

**Results:**

We have investigated the expression and function of miR-9 during early development of the mid-hindbrain region (MH) in chick. Our analysis reveals a closer relationship of chick miR-9 to mammalian miR-9 than to fish and a dynamic expression pattern in the chick neural tube. Early in development, miR-9 is diffusely expressed in the entire brain, bar the forebrain, and it becomes more restricted to specific areas of the CNS at later stages. MiR-9 overexpression at HH9–10 results in a reduction of *FGF8* expression and premature neuronal differentiation in the mid-hindbrain boundary (MHB). Within the midbrain miR-9 does not cause premature neuronal differentiation it rather reduces proliferation in the midbrain.

**Conclusion:**

Our findings indicate that miR-9 has regional specific effects in the developing mid-hindbrain region with a divergence of response of regional progenitors.

**Electronic supplementary material:**

The online version of this article (10.1186/s12861-017-0159-8) contains supplementary material, which is available to authorized users.

## Background

The development of vertebrate midbrain and anterior hindbrain depends on coordinated signals from the organiser region at the mid-hindbrain boundary (MHB). The MHB regulates the fate of neighbouring cells to adopt either a mesencephalic or metencephalic fate early in development [1–4], and experiments in chick embryos have shown that MHB transplants are able to induce mid-hindbrain fates in more anterior brain regions [5]. While, later in development, the MHB coordinates survival, cell proliferation, differentiation and migration [6]. The molecular basis of the organising activity of the MHB is mediated by a set of genes expressed anteriorly and posteriorly along the mid-hindbrain boundary. The morphogenes Wnt1 and Fgf8 together with transcription factors of the Pax and Engrailed (EN) family induce the formation of the MHB at the expression boundary of two other transcription factors, Otx in forebrain and Gbx2 in hindbrain [1, 7]. The subsequent interplay of Wnt1, Fgf8, Pax and En genes is critical for the maintenance of the MHB in later development [1, 6]. These MHB genes are found in all vertebrates that have been studied. However, subtle differences exist, e.g. the relative spatio-temporal onset of expression can differ between the species [3]. Many more genes are expressed in and around the MHB and have been shown to influence positioning, induction and maintenance of the MHB [6]. Failure of any of these leads to a variety of defects with profound implications for the organiser integrity like increased cell death, defective precursor cell proliferation, impaired differentiation and migration resulting in severe functional disruption of the MHB and thus development of midbrain and anterior hindbrain.

The territory in the immediate vicinity of the MHB is termed the intervening zone, IZ. This area has been shown to be necessary for the integrity of the MHB and different members of the Hes gene family are important for the maintenance of the IZ [8–11]. Furthermore, a marked feature of the IZ is that neurogenesis in this region is delayed compared to the surrounding domains [9, 11–14]. Thus, the mid-hindbrain (MH) domain is also characterized by a striking profile of neurogenesis.

A new player at the MHB is the microRNA-9 (miR-9). In zebrafish, Xenopus, and mouse miR-9 is expressed in regions adjacent to the MHB, but not in MHB [15–17]. Leucht et al., [15] showed that miR-9 is necessary to define the MHB. It targets the zebrafish fgf8 signaling pathway and restricts the IZ domain in the MHB. In zebrafish, Xenopus and mouse primary targets for miR-9 are members of the Hes gene family [15, 16, 18–20]. In turn, Hes1 in mouse, and her6 in zebrafish suppress miR-9 transcription [18, 19]. The IZ of zebrafish expresses her5, which is crucial for the formation of the IZ domain [9–11].

In this study, we investigated the role of miR-9 for the development of the chick MHB and midbrain. We have evaluated the evolutionary relationship of chick pre- and mature miR-9 with other vertebrates, and find that the miR displays high similarity to mammalian, and in particular to human miR-9 sequences. Our results showed that miR-9 is diffusely expressed in the brain early in development and becomes more focused later. Ectopic expression and suppression of miR-9 identified *FGF8* as in vivo target of miR-9. Ectopic miR-9 in the MHB resulted in premature neurogenesis in the IZ. Overexpression of miR-9 in the adjacent regions promoted neurogenesis only in hindbrain and not in midbrain. In midbrain we found that miR-9 is not expressed in dividing neural progenitor cells. Our results suggest miR-9 helps to define the extent of the MHB in chick, as shown in zebrafish [15] and can promote neurogenesis in MHB and hindbrain but not in midbrain.

## Methods

Fertilized Lohman and Hisex chicken eggs (LSL Rhein-Main, Geflügelvermehrungsbetriebe) were incubated in a humidified incubator at 37°C and embryos were staged according to Hamburger and Hamilton [21].

### Vector and oligonucleotide constructs

To introduce gga-miR-9 into the pSilencer U6.1 vector (Ambion), single stranded oligonucleotides containing a gga-miR-9 sense (5′-TCT TTG GTT ATC TAG CTG TAT GAT TCA AGA GAT CAT ACA GCT AGA TAA CCA AAG ATT TTTT-3′) and a miR-9 anti-sense (5′-AAT TAA AAA ATC TTT GGT TAT CTA GCT GTA TGA TCT CTT GAA TCA TAC AGC TAG ATA ACC AAA GAG GCC-3′) sequence were annealed and ligated into the ApaI/EcoRI digested pSilencer U6.1 vector (Ambion; miR-9-pSil). MiR-9 duplex was generated using RNA oligonucleotides (Ambion; Leucht et al. [15] with the sense - 5′ [UCU UUG GUU AUC UAG CAG AAU GA] _RNA_, and the antisense 5′ AUA CAG CUA GAU AAC CAA AGA]_RNA_ [TT]_DNA._ A control miR was generated using shuffled miR-9 sequence (Ambion; Leucht et al. [15]; sense - 5′-[UAU CAC UUC UAU AUG GUU UGG UG]_RNA_, antisense: 5′-[CCA AAC CAU AUA GAA GUG AUA] _RNA_ [TT]_DNA_). A locked nucleic acid (LNA) antisense inhibitor (miR-9-LNAi) (TCA TAC AGC TAG ATA ACC AAA G; Exiqon Product no. 427460–04) was employed to knock down mature miR-9 levels. To locate the electroporated cells the vector pCAX-EGFP [22] was co-injected (0.9:1 μg/μl; [22]). For target protection assays, single stranded DNA oligonucleotides to the target protections sites (Additional file 1: Figure S1G) were electroporated into the target region.

To validate miR-9 endogenous expression on a cellular level, the dual fluorescence reporter sensor (DFRS) vector developed by De Pietri Tonelli et al. [23, 24] was used. The green fluorescence protein (GFP) of the vector indicates all electroporated cells. The monomeric red fluorescence protein (mRFP) that is tagged by a 3’UTR complementary sequence of miR-9 indicates miR-9 activity: cells that express miR-9 silence mRFP. Control DFRS has a scrambled sequence tagged along with mRFP, therefore expresses continuously both, EGFP and mRFP.

### *In ovo* electroporation

Vectors or RNA- and DNA-oligonucleotides were diluted to a concentration of 1-2 μg/μl or 10 μM/25 μM, respectively, in 5% Fast green. Chick neural tubes were injected with Vector-DNA or oligonucleotides between HH9 and HH12 and midbrain was electroporated with platinum electrodes [25]. The transfected embryos were incubated for 1–3 days and fixed in 4% Paraformaldehyde (PFA).

### *Ex ovo* electroporation and time lapse

DFRS-S9 was electroporated *ex ovo* into HH10 midbrain and left for at least 4 h until GFP and RFP fluorescence were observed. Then the embryo was placed over an albumin-agarose gel in a petri dish and mounted under the confocal microscope. Pictures were taken every 30 min of at 543 nm and 488 nm for 13-27 h.

### Whole mount in situ hybridization

In situ hybridisations of mRNA (ISH) were performed as described [26]. For double in situ*,* embryos were first stained with BCIP/NBT and fixed in 4% PFA overnight before the second staining with either BCIP/INT or fast red was carried out. The following RNA antisense constructs were used: *FGF8* [27, 28], *WNT1* [29]*, NOTCH1* [30], *HAIRY1/HES1* [31] and *ENGRAILED (EN)* 1, *EN2* [32] and *PEA3* [33]. MiR-9 in situ hybridisation was performed with a customised antisense dig-miR-9-LNA oligonucleotide (locked nucleic acid, Product No. 70894, Exiqon) (LNA-ISH). Whole-mount embryos were hybridized with digoxygenin labelled miR-9 LNA (20-40 nM) over night at 45°C in a Chaps buffer (50% Formamide, 1.3% SSC, 5 mM EDTA, 50 μg/μl tRNA, 0.1% Tween 20, 0.1% CHAPS, 100 μg/μl Heparin, 2% Blocking reagent); then washed in a less stringent solution (2xSSC, 0.1% CHAPS) followed by low salt washes (0.2% SSC, 0.1% CHAPS). All other steps were carried out as in Li et al., [26].

### Immunohistochemistry

Whole-mount immunohistochemistry (IHC) was performed as previously described (Li et al., [26]) using antibodies against 3A10 (dilution according to batch; Developmental Studies Hybridoma Bank), Phosphp-Histone 3 (pH 3; 1:200; Millipore/Upstate) and GFP (1:1000; Polysciences). Appropriate Cy2 and Cy3 secondary antibodies (1:100 and 1:400 respectively; Dianova, Jackson ImmunoResearch) were applied to detect primary antibody binding. The slides or whole-mounts were post-fixed (4% PFA) and mounted in glycerol/PBS (9:1). Terminal desoxynucleotidyl transferase-mediated biotinylated UTP nick end labelling (TUNEL) assay was performed with the In Situ Cell Death Detection kit (Roche) according to the instruction of the manufacturer.

### Imaging

Sections and whole mounts were viewed and captured using a Leica M2FLIII stereomicroscope, a Zeiss LSM 510 META, Zeiss LSM5 Exiter and Zeiss Observer Z.1. The utilised imaging softwares were Axiovision 4.8, ZEN and Amira (trial version).

### Sequence collection and phylogenetic analysis of miR-9

Both mature and precursor sequences of miR-9 from diverse vertebrates (*Homo sapiens, Mus musculus, Rattus norvegicus*, *Gallus gallus, Taeniopygia guttata*, *Xenopus tropicalis*, *Fugu rubripes, Tetraodon nigroviridis, Danio rerio and Oryzias latipes*) were downloaded from miRBase Release 21:June 2014 [34]. Evolutionary relationship of miR-9 sequences was studied with the precursor sequences from vertebrates. The consensus structure of mature miR-9 was obtained using RNALogo [35]. Multiple sequence alignment was performed for mature and precursor sequences using MUSCLE 3.8 (http://www.ebi.ac.uk/Tools/msa/muscle/) with default parameter settings. The phylogenetic tree was constructed using the Neighbor-Joining algorithm in MEGA 6.06 [36]. The evolutionary distances were computed using the Maximum Composite Likelihood method. The phylogeny was tested by Bootstrap method with 1000 replicates.

### Computational analysis of miR-9 binding sites

Theoretical 3’UTR target sites of miR-9 were obtained from Target Scan (http://www.targetscan.org; [37–39]), and MicroCosm (http://www.ebi.ac.uk/enright-srv/microcosm/htdocs/targets/v5/; [40, 41]). Sequences of predicted target sites are listed in Additional file 1: Figure S1G.

## Results

### Chick miR-9 is evolutionary conserved

The miR-9 gene family has significantly expanded among vertebrates and consist of many variants. The number of miR-9 genes is in part a reflection of the whole genome duplication (WGD) events that have occurred during vertebrate evolution, but also due to more restricted gene duplications.

Multiple sequence alignment of pre-miR-9 showed a conserved region localised to the stem region of the hairpin structures (Additional file 2: Figure S2A), which leads to mature sequences (Additional file 2: Figure S2C). Variations in the sequences were observed in the flanking region of pre-miR-9. The evolutionary relationship of pre-miR-9 is shown in Additional file 2: Figure S2B. The phylogenetic analysis of precursor sequences showed that miR-9 of vertebrates clusters into three clades (Additional file 2: Figure S2B): clade I consists of miR-9-1 and miR-9-2, clade II contains miR-9-3 and miR-9-4, while clade III consists of miR-9 sequences from *Rattus norvegicus*. Clade I has subgroups I and II for miR-9-2 and miR-9-1, respectively. Subgroup I in clade I revealed that the miR-9-2 in Chicken and Zebra finch are more closely related with each other and with mammals than with fishes. Interestingly, miR-9-2 of *Gallus gallus* is more related to human miR-9-2 than to other mammals, which agrees with the International chicken genome sequence consortium [42], that reported that 60% of chicken genome is similar to human genes. The miR-9-2 variants of teleost fishes such as *Danio rerio, Fugu rubripes*, *Tetraodon nigroviridis* and *Oryzias latipes* are clustered as a distinct group. Intriguingly, miR-9-5 of *Danio rerio* is found closely related to miR-9-2 of mammals and chicken, whereas miR-9-2 of *Danio rerio* is closely related with miR-9-2 of teleost fishes. Subgroup II in clade I includes miR-9-1. The miR-9-1 of mammals forms one cluster along with *Xenopus tropicalis*, whereas teleost fishes clustered as a separate group. The variants miR-9-3 and miR-9-4 of mammals and teleost fishes formed clade II. Interestingly, miR-9-1 of *Gallus gallus* is found closely related with the miR-9-4 of *Danio rerio*.

Phylogenetic analysis also showed that mature miR-9 sequences are highly conserved [43, 44]. There are 21 conserved nucleotide positions across the diverse vertebrates. (Additional file 2: Figure S2C). The cladogram of mature miR-9 sequences revealed that chicken miR-9 is more closely related with the mammalian miR-9 (Additional file 2: Figure S2D). The miR-9 of fishes formed one cluster with amphibian. The consensus structure of mature sequences of miR-9 represents the degree of conservation in mature miR-9 sequences. The mature products from gga-miR-9-1 and gga-miR-9-2 have a similar sequences and are highly conserved (Additional file 2: Figure S2E).

### MiR-9 is expressed differentially along the mid-hindbrain area

**Fig. 1:**
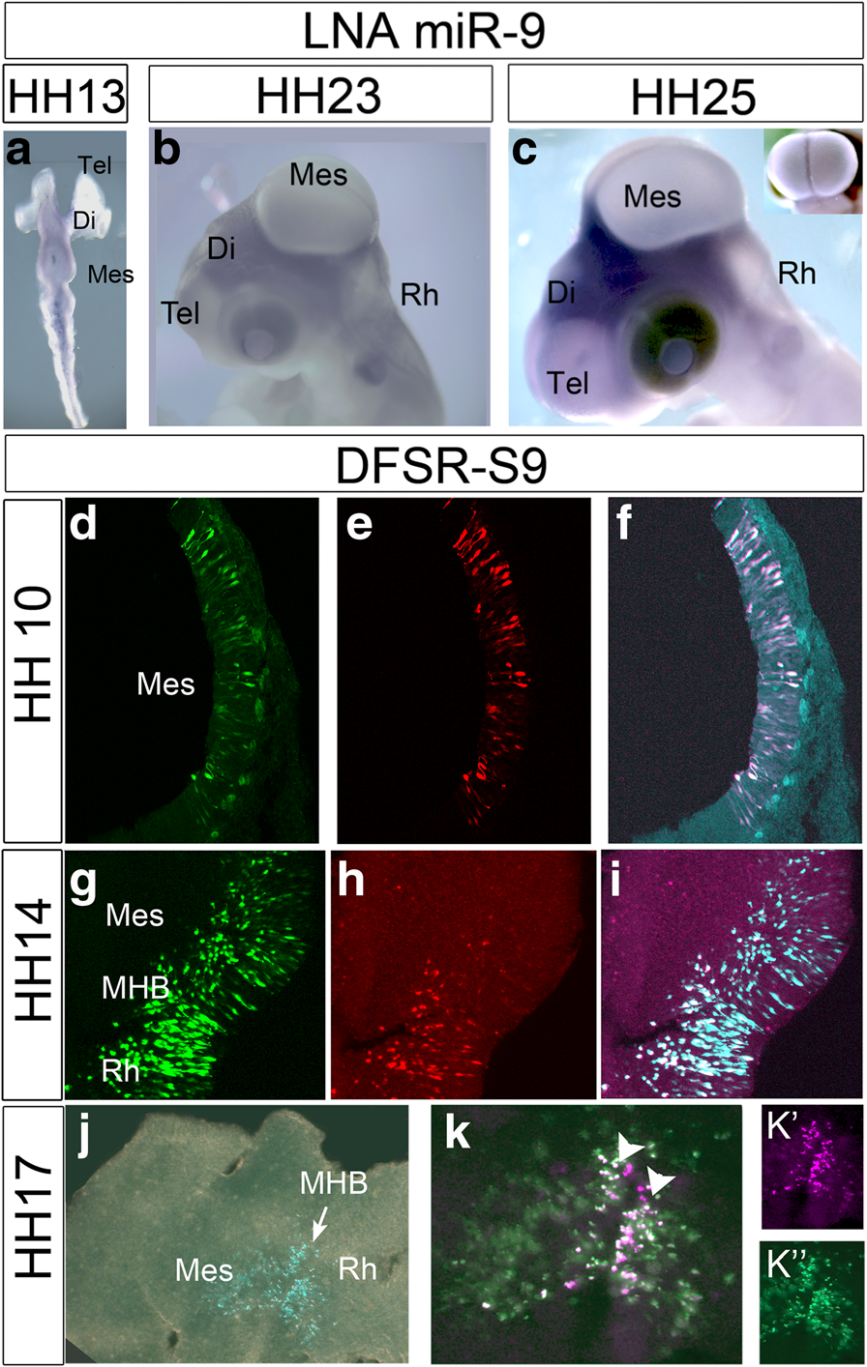
MiR-9 expression pattern in chick neural tube. LNA-ISH staining of whole-mount chick embryos (**a**-**c**) and miR-9 activity indicator DFRS-S9 (**d**-**k**”) showed that miR-9 is active from around HH13 in chick neural tube. LNA-ISH against miR-9 at HH13 (**a**) revealed an initially weak expression from di- to rhombencephalon. At HH23 (**b**) miR-9 was strong in ventral hind- and midbrain and in diencephalon. At HH25 (**c**) it was also expressed weakly in forebrain and spinal cord. The miR-9 sensor DFRS-S9 showed overlapping GFP (**d**) and RFP (**e**) positive cells in the mesencephalon at HH10 (**c**). At HH14 (**g**-**i**) not all GFP positive midbrain cells (**g**) expressed also RFP (**h**, **i**). At HH17 (**j**-**k**”) many cells in the mesencephalon were positive only for GFP (**j**-**k**”), whereas, cells in the MHB still expressed RFP (**k**-**K**″) and therefore no miR-9. Abbreviations: Di-diencephalon, Mes-mesencephalon, MHB-mid-hindbrain boundary, Rh-rhombencephalon, Tel-telencephalon

Next, we investigated the spatial and temporal expression of miR-9 in chick brain between embryonic day E2 (HH13) and E6 (HH29) (Fig. 1 and Additional file 3: Figure S3). Between E2 and E3 miR-9 was expressed in the chick brain from diencephalon to rhombencephalon (Fig. 1a-c; Additional file 3: Figure S3a-j; each stage *n* = 3 or more), and neither telencephalon nor spinal cord showed a strong expression (Fig. 1a). Horizontal sections revealed a general ubiquitous expression of miR-9 along the dorsoventral axis at E3 except for forebrain and spinal cord (HH18; Additional file 3: Figure S3A-F). Whole mount staining of E4 (HH 23) heads showed miR-9 strongly expressed in diencephalon, weakly in the telencephalon and not at all in dorsal midbrain (Fig. 1b). There was little change in expression at E5 except for stronger expression in the telencephalon (Fig. 1c). Horizontal sections through an E6 brain show miR-9 presence in the ventricular area of the neuroepithelium of posterior diencephalon, mesencephalon, rhombencephalon, and spinal cord (Additional file 3: Figure S3G-J), and in the mesenchyme surrounding the brain. This suggests that part of the strong staining visible in diencephalon of the entire embryo at E4 and later is due to mesenchymal labelling.

Since the weak and diffuse expression at E2 and E3 (Fig. 1a, Additional file 3: Figure S3A-F) in the neural tube did not reveal an obvious lack of miR-9 at the MHB as has been described in zebrafish, mouse and Xenopus [15–17], we employed another method to identify the activity of miR-9 at a cellular level. This method utilises a dual fluorescence vector (DFSR-S9; [24]) that reveals cells with active miR-9. In the absence of miR-9 the DFSR vector generates GFP and RFP. In the presence of miR-9, RFP, which contains a 3’UTR binding site for miR-9 is down regulated and only GFP is visible in the cell. We compared the DFRS-S9 GFP/RFP expression pattern along the MH with that of the control vector, which contains an arbitrary 3’UTR sequence after RFP, not complimentary to any known sequence (DFSR-Ctrl; [24]). We electroporated the vectors between HH9^−^-12 and fixed the brains after 5 h or 16 h. 5 h was the minimum time we found, after which, both GFP and RFP were expressed. Electroporation with DFSR-Ctrl showed a comparable expression of GFP and RFP in midbrain cells (Additional file 1: Figure S1C,F). At HH10 most of the DFRS-S9 expressing cells were positive for both GFP and RFP (Fig. 1d–f, *n* = 9/10). DFSR-S9 expression pattern revealed active miR-9 in the midbrain at around HH14 (E2) but not in the MHB and hindbrain (Fig. 1g-i; *n* = 8/9). At HH17 still a miR-9 free zone could be found in MHB in chick (Fig. 1j-K”, *n* = 3/3). The DFRS-S9 expression pattern suggests that miR-9 becomes active in the midbrain from about HH14 onwards but not in the MHB and not yet in the hindbrain.

### MiR-9 influences *FGF8* but not *HAIRY1* expression within the MHB

Our search for putative binding sites of miR-9 in the 3′ UTR of chick MHB core genes (Additional file 1: Figure S1G) yielded binding sites to the effector gene *FGF8*, the transcription factors *ENGRAILED* (*EN)* 1 and 2, and for *HES-1-B-like,* but none for the signalling protein *WNT1 or HAIRY1a/HAIRY1b/HES4*. To test whether any of these genes was regulated by miR-9 in vivo we performed miR-9 gain and loss of function experiments *in ovo*.

**Fig. 2:**
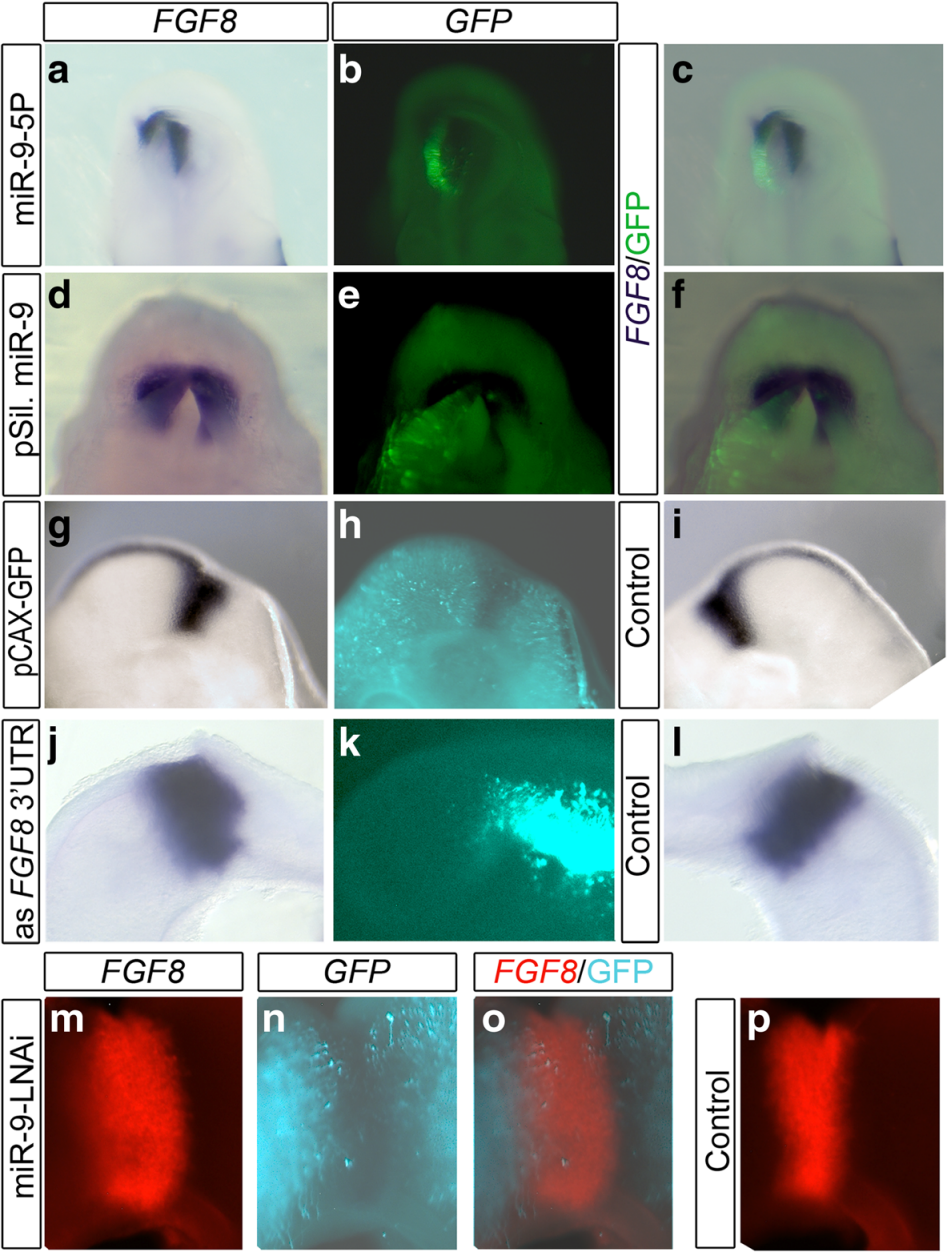
MiR-9 suppressed *FGF8*. **a**-**f** View from rhombencephalon onto the MHB (A-F) and lateral brain views (**g**-**p**). Left brain halves were electroporated at HH9/10 with miR-9 duplex (**a**-**c**), miR-9-pSil (**d**-**f**), pCAX-EGFP (**g**-**i**), and a single stranded antisense oligonucleotides against the 3’UTR FGF8 sequence (**j**—**l**). Right brain halves transfected with miR-9-LNAi (**m**-**p**)*.* The untransfected brain half served as control. ISH was employed to view *FGF8* mRNA (**a**,**c**,**d**,**f**,**g**,**i**,**j**,**l**,**m**,**o**,**p**), EGFP was immunostained with anti GFP (**b**,**c**,**e**,**f**,**h**,**k**,**n**,**o**). *FGF8* visualized with FAST RED achieved the red fluorescence in (**m**, **n**, **o**, **p**). **a**-**f** Overexpression of miR-9 reduced FGF-8 expression. EGFP overexpression had no influence on *FGF8* expression pattern (**g**-**i**). Ectopic expression of an antisense oligonucleotide to the 3’UTR binding site for miR-9 of FGF8 enlarged *FGF8* expression (**j**-**l**), as did the expression of miR-9-LNAi (**m**-**p**). Abbreviations: Mes-mesencephalon, MHB-mid-hindbrain boundary, Rh-rhombencephalon

We electroporated miR-9 as a duplex or a precursor form (pre-miR-9) for more immediate but shorter effects. For long lasting effects we overexpressed miR-9 stem loop via the pSilencer U6.1 vector (miR-9-pSil). All three constructs did result in miR-9 overexpression and down regulated RFP of the DFSR-S9 vector (Additional file 1: Figure S1B,E for miR-9-pSil, and data not shown). MiR-9 overexpression down regulated *FGF8* at the MHB (Fig. 2a-f). The strongest down regulation was seen when the embryos were electroporated at HH9/10 with the miR-9 duplex (Fig. 2a-c; *n* = 7/9). The effect of the pre-miR-9 (5/6) and of the miR-9 pSilencer (Fig. 2d-f; n = 7/11) was less prominent. Transfections at later stages (HH11–13, n = 7 for each stage; data not shown) or with pCAX-GFP (Fig. 2g-i; n = 9) did not result in an obvious down regulation. Collectively, only in 2 out of 21 brains transfected after HH10 *FGF8* expression seemed reduced. This result suggests that cells producing ectopic miR-9 are able to down regulate *FGF8* expression.

**Fig. 3:**
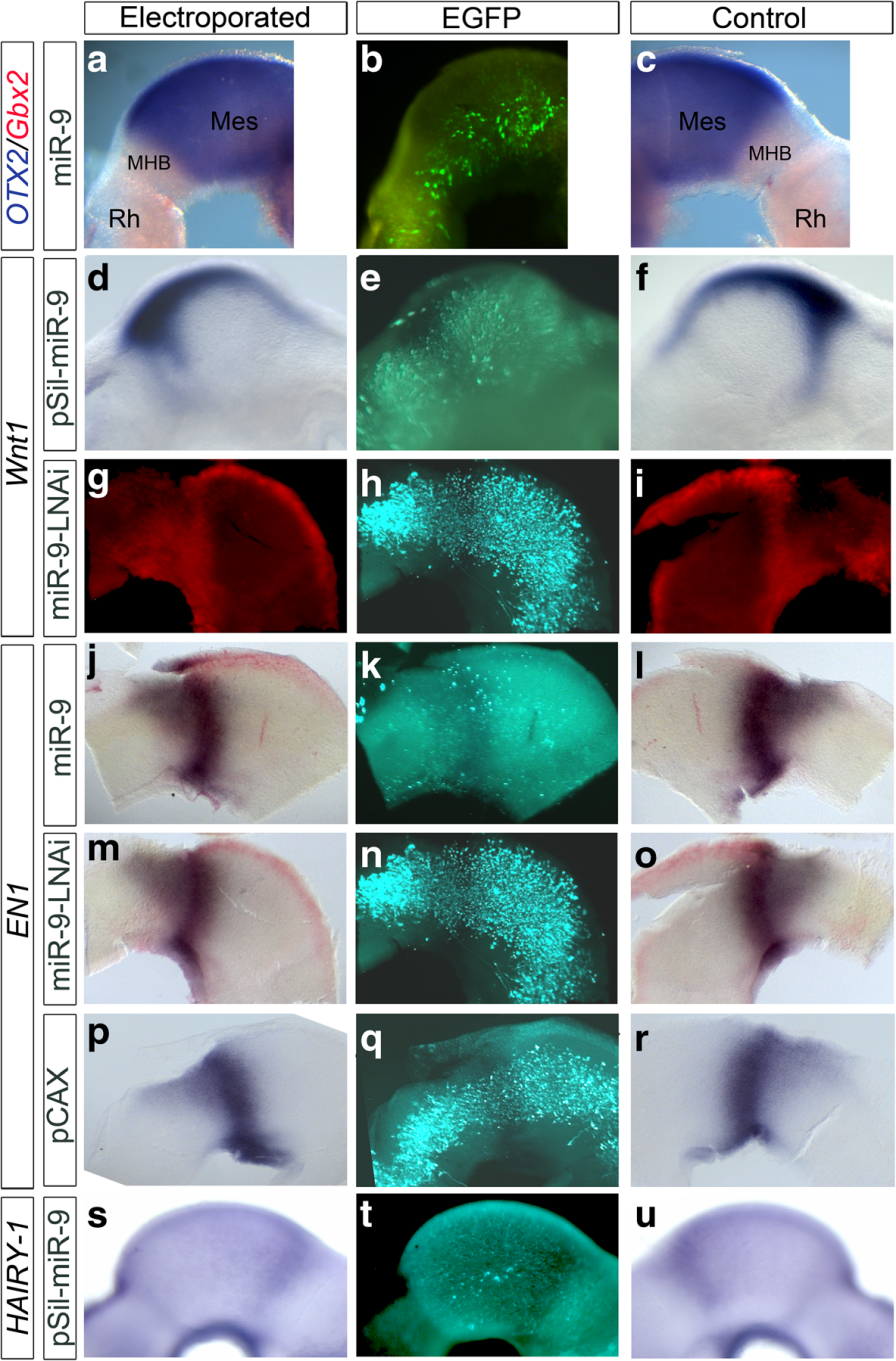
*OTX2, GBX2, WNT1* and *EN1* expressions were not disturbed by ectopic miR-9 expression. Lateral view of the neural tube at MH level overexpressing miR-9 (**a**, **b**, **d**, **e**, **j**, **k**, **m**, **n**, **s**, **t**), miR-9-LNAi (**g**,**h**,**m**,**n**,) or pCAX-EGFP (**p**,**q**). Left brain halves served as controls (**c**,**f**,**i**,**l**,**o**,**r**,**u**). Brains were electroporated at HH9–10 and fixed after 20-24 h. ISH for *OTX2* and *GBX2* (**a**, **c**), *WNT1* (**d**,**f**,**g**,**i**), *EN1* (**j**,**l**,**m**,**o**,**p**,**r**) and *HES1* (**s**,**u**). Electroporated cells were visualised with an immunostaining for EGFP (**b**,**e**,**h**,**k**,**n**,**q**,**t**). In (**g**,**i**) Fast Red was used to visualise *WNT1* with a red fluorescence. Ectopic miR-9 or miR-9-LNAi expression did not change the expression pattern of *OTX2* and *GBX2* (**a**), *WNT1* (**d**,**g**), *EN1* (**j**,**m**) or *HES1* (**s**). Abbreviations: Di-diencephalon, Mes-mesencephalon, MHB-mid-hindbrain boundary, Rh-rhombencephalon

To verify that miR-9 can affect Fgf8 expression at the MHB we knocked down miR-9 levels with miR-9-LNAi [18] at HH9/10. Reduced miR-9 activity resulted in an expansion of *FGF8* expression (Fig. 2m-p; *n* = 5/6). This result was further confirmed by ectopically expressing a *FGF8* 3’UTR target protection oligonucleotide along the MHB. Overexpression of *FGF8* 3’UTR target constructs also resulted in a broader *FGF8* expression compared to the control side (Fig. 2j-l; n3/4). These results show that FGF8 at the MHB is a direct target of miR-9 in chick a during early development. We next addressed whether miR-9 misexpression influences *PEA-3* expression as in zebrafish. Pea3 is expressed in the mid-hindbrain area and functions downstream of the Fgf8-MAPK signalling in zebrafish [45, 46] and chick [33]. Mir-9-5p overexpression at HH10 did not visibly reduce PEA-3 expression in midbrain at HH17 (*n* = 3; Additional file 4: Figure S4). The MHB is positioned at the expression interface of *Otx2* in midbrain and *Gbx1/2* in hindbrain [47–49]. We never observed any effect of miR-9 overexpression on the boundary between *GBX2* and *OTX2* (Fig. 3a-c; n = 3 for each, miR-9 duplex and miR-9-pSil). We also investigated if miR-9 influences *EN1* or *WNT1* expression along the MH. *WNT1* expression was only reduced in very few cases after ectopic miR-9 duplex expression (Fig. 3d-i; *n* = 4/15). The result is reminiscent of the indirect regulation of wnt1 in zebrafish [15] and agrees with the fact that there is no binding site for miR-9 in the 3’UTR of *WNT1*. Interestingly, we did not find an obvious down regulation of *EN1* expression along the MH area after overexpression of miR-9 at HH9/10 (Fig. 3j-l; n = 3/12). Similarly, ectopic miR-9-LNAi in the MH region caused no widening of *EN1* expression (Fig. 3m-o; *n* = 6/6). The loss and gain of miR-9 function resembled the control with ectopic EGFP-pCAX (Fig. 3p-r; n = 5). This result suggests a rather limited influence of miR-9 on *EN1* expression or one, which is not visible by ISH.

In zebrafish, miR-9 down regulated her5 in the MHB [15] and thus influenced the stability of the MHB by destroying the non-differentiating IZ. Therefore, we investigated whether overexpression of miR-9 has any influence on *HAIRY-1/HES1* [50] in the MH area. We never detected an obvious reduction of *HAIRY-1* expression in and around the MHB (Fig. 3s-u; *n* = 8/8). We concluded that miR-9 is not influencing *HAIRY-1* expression in the midbrain.

### MiR-9 expression promotes neurogenesis at the MHB and in anterior hindbrain

**Fig. 4:**
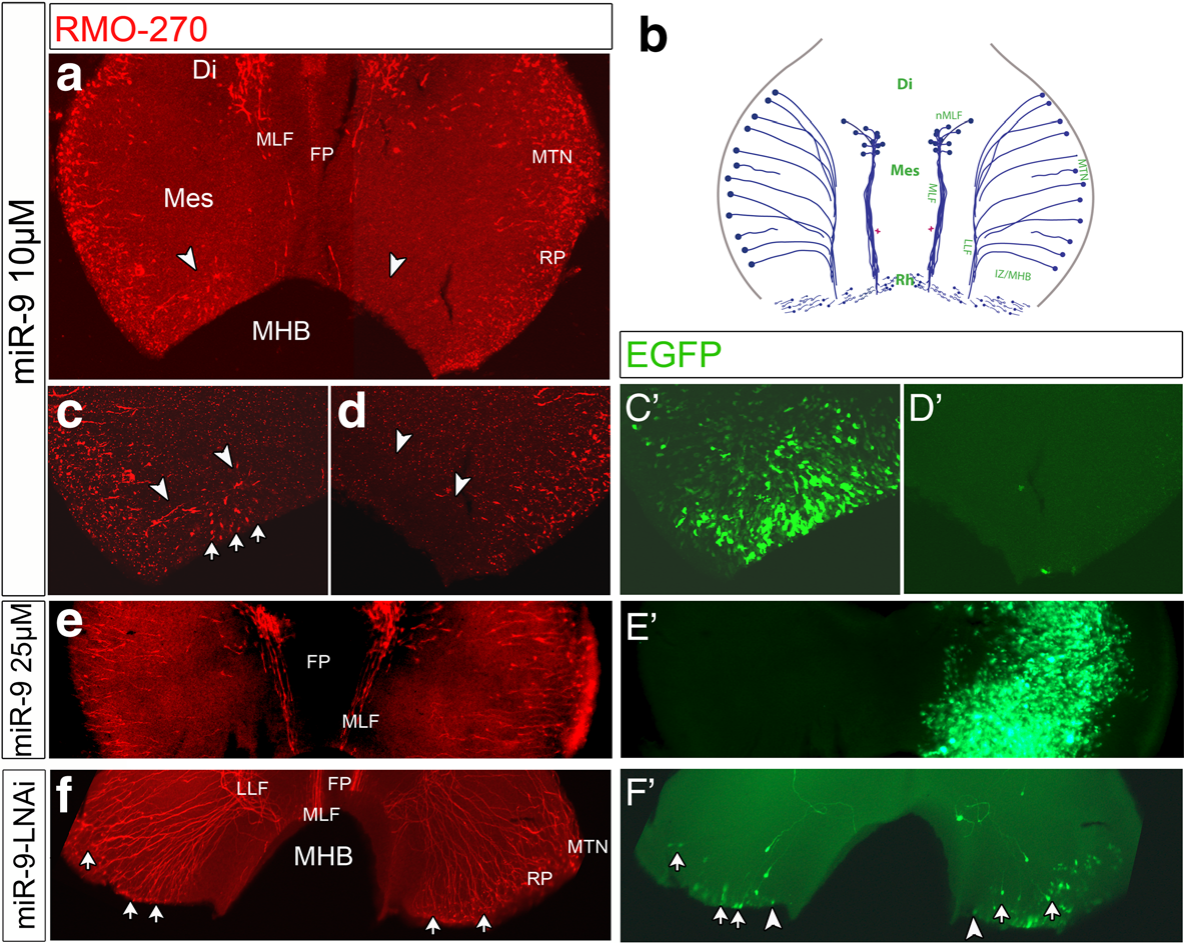
MiR-9 promoted neurogenesis in MHB and hindbrain. Open-book preparations of HH16/17 midbrains. Brain halves (**C**’,**E**’) or dorsal midbrain (**F**’) were electroporated at HH9–10 with miR-9 (**a**, **c**-**E**’) or miR-9-LNAi (**f**, **F**′). Transfected cells were immunostained for EGFP (green) and neurones and axons for 3A10 (red). **b** is a scheme of the specific pattern of neurogenesis in midbrain around HH16/17. Overexpression of miR-9 did not promote ectopic neurogenesis within the midbrain (**a**, **c**-**E**’) but in the MHB (**a**,**c**). (**c**-**D**’) are magnifications of the MHB area in (**a**). The white arrowheads in (**a**) to (**d**) indicate the posterior border of the mesencephalon and the beginning of the IZ of the MHB. Ectopic miR-9 in the left half of the MHB (**a**, **c**) induced ectopic neurones (white arrows), which are not found in the control right half (**a**, **d**). Ectopic miR-9 in posterior midbrain (**a**, **c**,**C**’) or in more anterior midbrain regions (**e**,**E**’) did not promote early neurogenesis in midbrain. Some of the miR-9-LNAi expressing cells in dorsal midbrain (**f**-**F**′) developed axons (arrows). MiR-9-LNAi expressing cells in the MHB did not show long axons (arrowheads in **F**′). Abbreviations: Di-diencephalon, FP-floor plate, IZ-intermediate zone, Mes-mesencephalon, MHB-mid-hindbrain boundary, MLF-medial longitudinal tract, MTN-mesencephalic trigeminus nucleus, nMLF – nucleus of MLF [82], LLF-lateral longitudinal tract, Rh-rhombencephalon, RP-roof plate

**Fig. 5:**
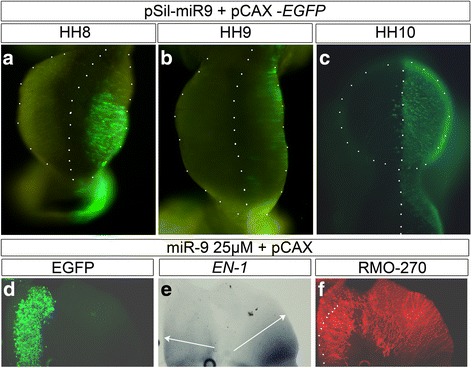
MiR-9 reduced size of midbrain. Broad overexpression of miR-9 along the DV axis of the midbrain result often in smaller midbrains (15/18). Transfection of pSil-miR-9 (**a**-**c**) in right brain halves at HH8 (**a**), HH9 (**b**), HH10 (**c**) reduced the size of midbrain halves compared to left control side. (**d**-**f**): Open-book preparation of midbrain with ectopic miR-9 duplex from HH8 shows a reduced growth of the electroporated side (**d**-**f**) and a weakened expression of *EN1* (**e**). 3A10 immunostaining showed a relatively normal pattern of neurogenesis only compressed into a smaller area (**f**). Abbreviations: FP-floor plate, Mes-mesencephalon, MHB-mid-hindbrain boundary, Rh-rhombencephalon, RP-roof plate

In zebrafish and Xenopus miR-9 overexpression promoted neural differentiation in the hindbrain and in zebrafish also in the MHB [15, 16, 19]. In mouse, zebrafish and Xenopus miR-9 affected either her5 (in zebrafish MHB) or Hes1 in the mouse telencephalon and Xenopus hindbrain [15, 16, 18, 20, 51]. Our ectopic expression of miR-9 did not result in an obvious down regulation of *HES1* (Fig. 3s-u, see above) within the MH area. However, overexpression of miR-9 duplex at HH9–10 promoted ectopic neurones within the MHB (Fig. 4a, C-D’; n = 6/8) and in the anterior rhombencephalon (Additional file 5: Figure S5; n = 4/5) but not in the rest of the midbrain (Fig. 4a, c-E’; *n* = 19/20). Brains electroporated with miR-9-LNAi at HH9–10 did not show an obvious loss of differentiated neurones in the dorsal midbrain, where the mesencephalic trigeminal nucleus (MTN) is generated (Fig. 4b, f, F’; *n* = 0/11). However, the transfected neurones showed long axons comparable to the ‘wildtype’ neurones surrounding them (compare Fig. 4f and F’ and white arrows). Interestingly, the miR-9-LNAi-GFP+ cells within the MHB did not sprout axons or label as neurones (white arrowhead in Fig. 4F’). This result suggests that within the MHB and the anterior hindbrain miR-9 can promote neurogenesis but not in midbrain. Although ectopic expression of miR-9 at HH9–10 did not result in ectopic neurogenesis within midbrain areas (Fig. 4), we detected that a broad overexpression of miR-9 reduced the size of the transfected midbrain compared to the uninjected control site Fig. 5a-c, n=26/42). This effect was not observed when EGFP alone was transfected into midbrain (data not shown). Ectopic expression of high concentration of miR-9 (25 μm) at HH8 resulted in an even more obvious reduction of midbrain size (Fig. 5d-f). In this case *EN1* expression was also reduced (Fig. 5e). We never observed ectopic neurones in E3 (HH17–19) midbrains but at HH26 (E5) more cells transfected with pSil-miR-9 seemed to have located to the mantle zone than cells expressing pCAX-GFP (Additional file 7: Figure S7H,I).

We next investigated whether miR-9 overexpression caused apoptosis or affected the number of cells undergoing mitosis. Tunnel staining did not indicate additional apoptotic cells in midbrains independent of their transfection with miR-9-5p, miR-9-LNAi or pCAX-GFP (Additional file 6: Figure S6). However, the number of phospho-histone-H3 positive cells (pH 3+) also expressing miR-9 (n=4), miR-9-LNAi (n=2) or pCAX-GFP (n=2) at E3 differed (Additional file 7: Figure S7). An average of 15% of pH 3+ cells also expressed miR-9 compared to 51% GFP+/pH 3+ cells in control midbrains (Additional file 7: Figure S7 J,K). The result suggests that miR-9-5p positive cells are around 60% less likely to express pH 3 (Additional file 7: Figure S7 K). Interestingly, miR-9-LNAi overexpressing midbrains cells were also less likely to co-express pH 3 but showed in general a low amount of pH 3 positive cells. Smaller midbrain halves and less mitotic miR-9+ cells suggest that although miR-9 does not promote neurogenesis in early midbrain, it can limit progenitor proliferation.

### MiR-9 is downregulated in dividing cells

**Fig. 6:**
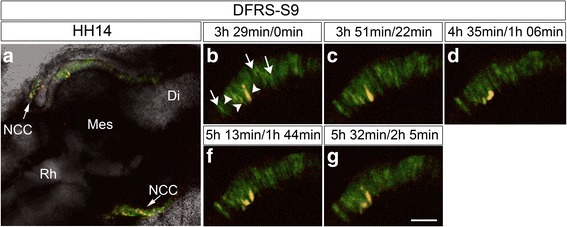
Dividing mesencephalic progenitor cells did not express miR-9. The right half of the mesencephalon was electroporated with miR-9 sensor DFRS-S9 ex ovo at HH10. **a** shows a dorsal view the midbrain at HH14. Both, midbrain cells (in box) and NCC (arrows) were transfected. (**b**-**f**) are magnifications of the marked area in (**a**) and show the process of a cell division. The time distance between the pictures is indicated above in the time within the video and the absolute time for the cell division. Several of the neuroepthelial cells express only GFP (white arrows) some express GFP and RFP (white arrowheads). **b**-**f** show a dividing cell that expressed both, GFP and RFP and thus no miR-9. Abbreviations: Di-diencephalon, Mes-Mesencephalon, NCC-neural crest cells, Rh-rhombencephalon; scale bar 100μm


To investigate in vivo whether progenitors or differentiating neurones, or both, normally express miR-9 we electroporated the miR-9 detector vector DFRS-S9 [24] into midbrain and established a time-lapse procedure for the chick brain (procedure will be published elsewhere). Our time-lapse experiments showed that dividing progenitors in the ventricular zone of the midbrain do not express miR-9 at HH12. We also observed that in the adjacent regions, the posterior diencephalon and anterior hindbrain. The dividing cells expressed both, EGFP and RFP at HH12 (Fig. 6 and Additional file 8: Movie S1). Cells in the mantle zone however, and some cells on their way to the mantle zone expressed only EGFP, and therefore express miR-9 (Fig. 6 and Additional file 8: Movie S1). Taken together, these results suggests in midbrain miR-9 is excluded from dividing neural progenitors cells.

Additional file 8: Movie S1.Mitotic cells were devoid of miR-9. The movie was generated from a time-lapse confocal microscopy study using Zen 2012 (Zeiss Inc). Midbrain was transfected with DFRS-S9 ex ovo and time lapsed for 6 h, with pictures taken all 10 min beginning at late HH13. The cells on the top are neural crest cells and the bottom cells locate to in midbrain neuroepithelium. The cell division observed in the movie took approximately 3 h time. GFP^+^/RFP^+^ cells are devoid of endogenous miR-9 whereas, GFP^+^/RFP^−^ cells produce miR-9. There are several other GFP^+^/RFP^+^ visible, which very likely are mitotic cells. The movie shows dividing neural progenitor cell lack miR-9. (AVI 2000 kb)


## Discussion

In this study we have shown that chick miR-9 forms part of an evolutionary conserved family. Our findings further suggest that miR-9 has different roles in midbrain, MHB, and hindbrain. In the MHB and the hindbrain ectopic miR-9 can inhibit FGF8 signalling by down regulating *FGF8* and promotes neurogenesis. In midbrain expression of miR-9 reduced proliferation; but did not promote neurogenesis early in development.

### Evolutionary conservation of chick miR-9

MiR-9 is one of the ancient miR, which appeared with the bilateria, and the miR-9 subfamily is evolutionarily conserved among vertebrates [43, 44]. In mammals, *Homo sapiens* and *Mus musculus* have three variants (miR-9-1, miR-9-2, miR-9-3) whereas *Rattus norvegicus* displays six miR-9 genes. The amphibian, *Xenopus tropicalis* has four variants. In teleost, *Danio rerio* and *Oryzias latipes* have seven variants whereas, *Fugu rubripes* and *Tetraodon nigroviridis* have four variants. These numbers undoubtedly reflect, in part, the influence of whole genome duplications (WGD). Two rounds of WGD are believed to have have occurred at the origin of jawed vertebrates and an additional teleost specific WGD has been observed [43]. Only two variants (miR-9-1 and miR-9-2) of miR-9 are found in birds, such as *Gallus gallus* and *Taeniopygia guttata*. In chick, both the variants (gga-miR-9-1 and gga-miR-9-2) produce the same mature product, miR-9-5p. The difference in the number of gene families are due to a substantial reduction in interspersed repeat content, pseudogenes and segmental duplication within the chicken genome. *X. tropicalis* e.g. retained all the miRs, *H.sapiens* only three [43]. In most species both strands of miR-9 variants (guide- and passenger strand) participate in gene regulation, thus the number of miR-9s is even higher [43, 52].

Our phylogenetic tree showed gga-miR-9-2 is more related to human miR-9 than to other vertebrates and gga-miR-9-1 is closely related with the dre-miR-9-4. This is different from the phylognetic relations presented in Yuva-Aydemir et al. [44], which is based on miR sequences from an earlier release of miRbase (release 16 versus 21). In addition, we only compare vertebrate miR-precursors, whereas Yuva-Aydemir et al. [44] also included invertebrate sequences.

### Divergent expression of miR-9 in brain and spinal cord

The spatial and temporal miR-9 expression pattern we observed differs slightly from that described by Darnell et al. [53]. Darnell et al. [53] described first miR-9 expression at HH22 with a strong expression in forebrain. We see observed first expression in telencephalon a stage later but general miR-9 expression already at HH13. The difference in the intensity of the forebrain staining might be due to differences in stage or chick strains. To confirm the early expression of miR-9 in the mid-hindbrain area, we used a miR-9 reporter sensor vector (DFRS-S9, [24]). At HH10 no active miR-9 was observed, but three stages later the miR-9 sensor showed miR-9 positive cells in midbrain but not in MHB. Thus, our results agree with findings in mouse, Xenopus and zebrafish, which show that the MHB lacks miR-9 expression [15–17, 19]. In zebrafish, mouse and Xenopus miR-9 expression begins in the telencephalon and spreads posteriorly to the rest of the brain and to the spinal cord in mouse and zebrafish. We observed miR-9 expression first in posterior brain. Later, miR-9 localises to the ventricular zone like in other vertebrate brain regions [16, 17, 19]. Taken together, in chick miR-9 is lacking in MHB.

### MiR-9 effect on MHB genes

Our target analysis scan suggested 3’UTR seed sequences for miR-9 with *FGF8*, *EN1* and *EN2*. In contrast to zebrafish, human, mouse and Xenopus En1 and En2 also possess a possible 3’UTR seed region for miR-9 (data not shown). Overexpression or reduction of miR-9 at HH9/10 (E1.5) in MH resulted in a down- or up-regulation of *FGF8*, respectively but no obvious changes in *EN1* and *EN2* expression. *EN1* and *EN2* are two of the core genes that maintain the MHB [54] and their loss results in reduced cerebellum and midbrain [54, 55]. We found one very obvious down regulation of *EN1* expression. However, in this case the entire half of the brain was smaller (Fig. 5). Thus, we are not able to exclude that because of the smaller brain area *EN1* expression appears weaker. *FGF8* expression was also not visibly changed by miR-9 transfections at later stages. At HH10 the MHB just has formed and thus might be more susceptible to influences than later in development. Accordingly, miR-9 did not interrupt the sharp boundary between *OTX2* and *GBX2*, which is set up earlier. At later stages the other core proteins at the MHB (e.g.WNT1, EN1, EN2, PAX7) balance FGF8 expression much better than right after boundary formation [6].

Thus, our miR-9 misexpression experiments interfered with proteins after MHB has formed. The time-limited sensibility for miR-9 influencing *FGF8* is supported by the lesser effect of pre-miR-9 and miR-9-pSil on FGF8 expression we observed. In both cases mature miR-9 has to be generated first whereas, mature miR-9-5p can be active immediately after electroporation. Pre-miR-9 might also have had less effect since endogenous pre-mir-9 hairpin sequences not only generate mature miR-9 but also antisense i.e., miR-9* [56]. In chick we electroporate and analyse at specific time points and in specific areas. In zebrafish and Xenopus overexpression or down regulation of miR-9 began already at the 2-cell stage [15, 16]. The change of miR-9 and its impact on the MHB in zebrafish and on neural development in zebrafish and Xenopus may therefore reflect an impact of miR-9 very early in development, whereby both sides of the brain are affected. We only transfect one half and have the other as control and never observed a loss of the MHB area. In zebrafish, Leucht et al. [15] described pea3, a downstream target of fgf8 as additional target of miR-9. Our target analysis scans did not suggest chick *PEA3* as a direct target of miR-9 and we did not observe a visible downregulation of its expression in the MH 24 h after miR-9 overexpression. We suspect that PEA3 will be indirectly downregulated like *WNT1* and any effect would be seen later. Nevertheless, in chick midbrain Pea-3 has been shown to regulate the size of the MHB [33]. Overexpression of PEA-3 in midbrain results in a larger MHB and tectum and not a fate transfer into hindbrain as ectopic Fgf8 provokes. Thus, PEA-3 is a likely candidate to be affected and will very likely be downregulated visibly at later stages.

In Xenopus, miR-15/17 is necessary to sharpen the Nodal gradient [57, 58]. Our results in chick suggested that miR-9 around the MHB might sharpen the *FGF8* gradient in this area and thus very likely helps to sustain the MHB area and its size.

### MiR-9 inhibits proliferation in midbrain but not neurogenesis

MiR-9 is known to influence the balance between proliferation and differentiation of neural progenitors and neurones in several systems and species [15–18, 59, 60]. We observed smaller midbrain halves after overexpression of miR-9 in a very broad area 24 h after electroporation. Interestingly, this effect was not accompanied by premature neurogenesis. This suggests that in midbrain miR-9 can inhibit proliferation but does not necessarily promote neurogenesis during early development. Our results correlate with those of other vertebrates where one fundatmental function of miR-9 is to inhibit neural proliferation [15–18, 60–64].

In zebrafish hindbrain, miR-9 has been described to have a twofold effect. It inhibits members of the hes family that promote proliferation, and it also blocks elavl3 that advances neuronal differentiation and blocks proliferation [19, 65]. Interestingly, both, decrease and increase of miR-9 showed this effect. MiR-9 knockdown [43] leads to a disinhibition of her6 and elavl3, which results in more cycling progenitors and committed neurones. Overexpression of miR-9 leads to many committed neural cells since her6 is blocked and to many radila glia cells because elavl3 is reduced [65]. The net effect of miR-9 is to keep cells in the ambivalent state of neural progenitors by accumulating in the cell. To promote differentiation versus proliferation the main target of miR-9 are Hes genes [15, 16, 18–20, 66]. They regulate the maintenance of neural progenitor cells by repressing proneural gene expression [67]. Mature miRs are robust molecules and some can have a half life of days [68]. Very likely, mature miR-9 accumulates in a cell and slowly reduces Hes1 [65] and hence contributes to the oscillation of Hes1 as Bonev et al. (2012) [18] have described in vitro.

Our theoretical target scan suggested a 3’UTR seed site of miR-9 for *HES-1-B-like* but not for *HES1/HAIRY1A/HES4* in chick. Nevertheless, since *HES1* is a miR-9 target in other vertebrates we examined HES1 expression after miR-9 overexpression. *HES1* is expressed at HH10 along the entire mid-hindbrain region [5, 30] and gives rise to a stronger expression at the boundary of di- and mesencephalon and the anterior MHB from HH14 to HH16/17. Ectopic miR-9 expression did not change *HES1* expression pattern visibly, nor did overexpression of miR-9-LNAi to block miR-9 or in vivo target protection. It might be that on a single cell level a change could be seen or that there are other regulatory factors involved at these stages. For example, there are other members of the HES gene family expressed along the mid-hindbrain. *HES5* is found in the entire CNS with different expression levels at HH9 [69]. At HH11 and 14 it is expressed strongly in the entire CNS except for the MHB [69, 70]. *HES6* is strongly expressed in anterior hindbrain but weakly in the rest of the brain [71]. The presence of *HES5* and *HES6* in chick midbrain could be responsible for the lack of premature neurogenesis we observe after miR-9 overexpression. This result is reminiscent to the loss of Hes1 in mouse cortex, where the absence of Hes1 or Hes5 alone causes minimum defects [72–74], while the absence of both results in premature exhaustion of neural precursor cells and accelerated neurogenesis [74].

### MiR-9 promotes neurogenesis in the MHB and hindbrain

The expression of miR-9 in neural progenitors in the CNS of chick, mouse, xenopus and zebrafish [15–17, 20, 53, 75, 76] and the results of experimental over- and down regulations [15–18, 60, 63] suggest that in most brain regions miR-9 promotes neuronal differentiation. We showed that miR-9 overexpression in chick MH also generated ectopic neurones in the anterior hindbrain and the long lasting progenitor region of the MHB (IZ) but not in the midbrain.

Chick midbrain shows a very specific neuronal differentiation pattern. At early stages the only neurones that differentiate belong to mesencephalic trigeminus nucleus (MTN, [77]; see scheme in Fig. 4). Hence, midbrain might express additional regulators of neurogenesis compared to the rest of the MH. Several mechanisms could be responsible for the lack of ectopic neurogenesis in miR-9 transfected midbrains cells. MiR-9 might not be sufficient to cause neural differentiation or specific factors, only present in midbrain, might regulate miR-9 or prevent premature differentiation. All these regulations have been reported. The conversion of human neonatal fibroblasts into neuronal cells needs three different miRs [78]. Transcription factors like Hes1 or TLX can inhibit miR-9 [18, 64, 79] and DNA binding proteins and FXFRqP genes are known to influence miR-9 regulation [80, 81]. In addition, other miRs might interact to set a steady state of proliferation and differentiation. MiR-107 for example, inhibits dicer protein in zebrafish hindbrain, which normally activates miR-9 biogenesis and thus differentiation [65].

In zebrafish hindbrain and mouse telencephalon miR-9 is expressed in a range of progenitor cells with different commitments [18, 19]. In the cortical ventricular zone miR-9 was high in cells with low Hes1 and vice versa and it was present in some of the differentiating neurones in the mantle zone. In zebrafish hindbrain non-cycling progenitors and differentiating neurones expressed miR-9 [43]. In early chick midbrain miR-9 expressing cells might also belong to non-dividing progenitors. MiR-9 positive cells were less likely to be mitotic and were never observed to divide in the time-lapse study. Interestingly, at later developmental stages more miR-9 expressing cells are observed in the mantle zone.

## Conclusion

Our results support the theory of a tissue specific regulation of miR-9 and multiple roles during neuronal development. MiR-9 overexpression caused premature neurogenesis in MHB and hindbrain but not in midbrain. In early midbrain our results support a role of miR-9 in regulating proliferation and hence midbrain size. This effect could be direct on suppressing proliferation or indirect by suppressing FGF8 signalling.

## Additional files

Additional file 1: Figure S1. Functional miR-9 overexpression. (A) depicts wildtype cells expressing GFP after transfections with DFRS-S9 sensor plasmid. The red, RFP expressing cells in (D) are those cells of (A) that do not express miR-9. Overexpression of miR-9 together with DFRS-S9 (B,E) showed that almost all cells in midbrain express only GFP (B) and no RFP (E) and therefore miR-9. With the DFRS-control sensor plasmid all transfected cells express GFP (C) and RFP (F). (G) shows the binding sequences for miR-9 in the 3’UTRs of *FGF8*, *EN1*, *EN2* and *HES1-B-like*. Abbreviations: Di-diencephalon, Mes-mesencephalon, Rh-rhombencephalon. (PNG 3031 kb)

Additional file 2: Figure S2. A. CLUSTAL multiple sequence alignment of pre-miR-9 sequence by MUSCLE (3.8) shows that throughout vertebrate species miR-9 is conserved in the stem region of the hairpin (indicated by blue letters and asterix marks). B. Evolutionary relationship of pre-miR-9 family. The Neighbour-Joining phylogram shows the phylogenetic relationships of pre-miR-9 variants among vertebrates. The evolutionary distances were computed using the Maximum Composite Likelihood method and the branch lengths are proportional to the number of base substitutions per site. The scale bar indicates substitution rate of nucleotides per site. Evolutionary analysis was performed in MEGA6. The colour denotes the Clades of miR-9 family in vertebrates (Clade I: red, Clade II: Rose and Clade III: Blue). Clade I has two subclades, namely miR-9-1 and miR-9-2. Clade II has miR-9-3 and miR-9-4. Clade III consists of miR-9 from Rattus norvegicus. C. CLUSTAL multiple sequence alignment of mature miR-9 sequence by MUSCLE (3.8). The conserved sequences are indictaed in pink and with asterix marks. D. The cladogram shows the evolutionary relationship of mature miR-9 among different vertebrates. Branch lengths are not proportional to the sequence divergence. E. The stem loop structure of Gallus gallus miR-9 variants. The gga-miR-9-1 gives raise to functional mature miR-9 (miR-9-5p) and miR-9* (miR-9-3p), while gga-miR-9-2 only produce miR-9 (shown in colour). The mature miR-9 obtained from both gga-miR-9-1 and gga-miR-9-2 has the same sequences and evolutionarily conserved. (PDF 369 kb)

Additional file 3: Figure S3. Expression of miR-9 in HH18 and E6 chick brains. Coronal sections through HH18 (A-F) and HH26 (E6; G-J) chick brains. (A-F) are sections on the level of forebrain (A), mesencephalon (B,C) rhombencephalon (D), posterior rhombencephalon (E) and spinal cord (F). (G-J) are sections through di- and mesencephalon (G,H), rhombencephalon (I), and spinal cord (J). Note, miR-9 expression in the ventricular zone of HH26 diencephalon, mesencephalon and rhombencephalon. Abbreviations: De-dermamyotome, Di-diencephalon, Mes-mesencephalon, NC-notochord, oV-otic vesicle, Rh-rhombencephalon, Sc-sclerotome, Te-tectum. Scale bars-100 μm. (PNG 6983 kb)

Additional file 4: Figure S4. *PEA3* expression is unchanged by miR-9 overexpression. Lateral view of the MH level. Left brain half was electroporated with miR-9 duplex (A) at HH10. The untransfected brain half served as control (B). The insert in (B) shows the transfections. *PEA3* (blue) was visualised by ISH. (PNG 1230 kb)

Additional file 5: Figure S5. Overexpression of miR-9 in anterior hindbrain promotes neurogenesis. MiR-9 (pSil-miR-9) was ectopically expressed in anterior hindbrain at HH9 (A,B) and HH11 (C,D) in left (A,B) or right (C,D) brain half. The other brain half was used as control. The white arrowheads in (B) point to ‘ectopic’ neurones in rhombomere 1, which are not present in right rhombomere 1 (arrows). Overexpression of miR-9 at later stages (C; HH11) did not result in early neurogenesis in anterior hindbrain (white arrowheads, D). Abbreviations: Rh-rhombomere, FP-floor plate. (PNG 3150 kb)

Additional file 6: Figure S6. MiR-9 does not raise apoptosis. Section of HH17 midbrains overexpressing pCAX-EGFP (A,B), miR-9-5p (C,D) or miR-9 LNAi (E,F). (G,H) are wild type sections treated with DNase (G) to evoke a positive reaction of Tunnel staining in cells. The arrow in the magnified insert in (G) shows the black Tunnels stained cell nuclei after DNase treatment. No Tunnel staining was observed in the electroporated and non-electroporated midbrain halves (A-F). Scale bare: 200μm. (PNG 7740 kb)

Additional file 7: Figure S7. MiR-9 misexpression and mitotic cells. Section of HH17 midbrains overexpressing pCAX-EGFP (A,A’,E,E’,E”’,I), miR-9-5p (B,B’F,F′,F″) or miR-9 LNAi (C,C’,G,G’,G”). Sections of HH26 midbrain transfected with pCAX-EGFP (I) and pSil-miR-9 (D,D’,H). Sections were immunostained for EGFP (green) and pH 3 (red). (A-C, E”-G”) show the overlays of GFP and pH 3 expressing cells. (E-G”) are magnifications of (A,B,C), respectively and (H) is a magnification of (D). Several EFGP+/pH 3+ cells are indicated by arrowheads in (E”) and (F″). The magnifications in (H) shows more GFP+ cells in the mantle zone than the control (I). (J,K) are percentage graphs displaying the percentage average of GFP+, pH 3+ and GFP+/pH 3 cells after different treatments of midbrain. (K) shows the percentage of pH 3+/GFP+ cells of pH 3+ cells. Midbrain cells expressing only GFP are almost two thirds more likely to express pH 3. Scale bars in (C’, I): 100 μm; scale bare in (D’): 200 μm. (PNG 7500 kb)

## Abbreviations

Di: diencephalon
FP: floor plate
IZ: intermediate zone
LLF: lateral longitudinal tract
Mes: mesencephalon
MHB: mid-hindbrain
MLF: medial longitudinal tract
MTN: mesencephalic trigeminal nucleus
nMLF: nucleus of MLF
Rh: rhombencephalon
RP: roof plate
Tel: telencephalon
