# Supplementary figures and images for: Expression and function of microRNA-9 in the mid-hindbrain area of embryonic chick
Source: BMC Dev Biol. 2018 Feb 22;18:3. doi: 10.1186/s12861-017-0159-8 (PMC5824543; doi:10.1186/s12861-017-0159-8)

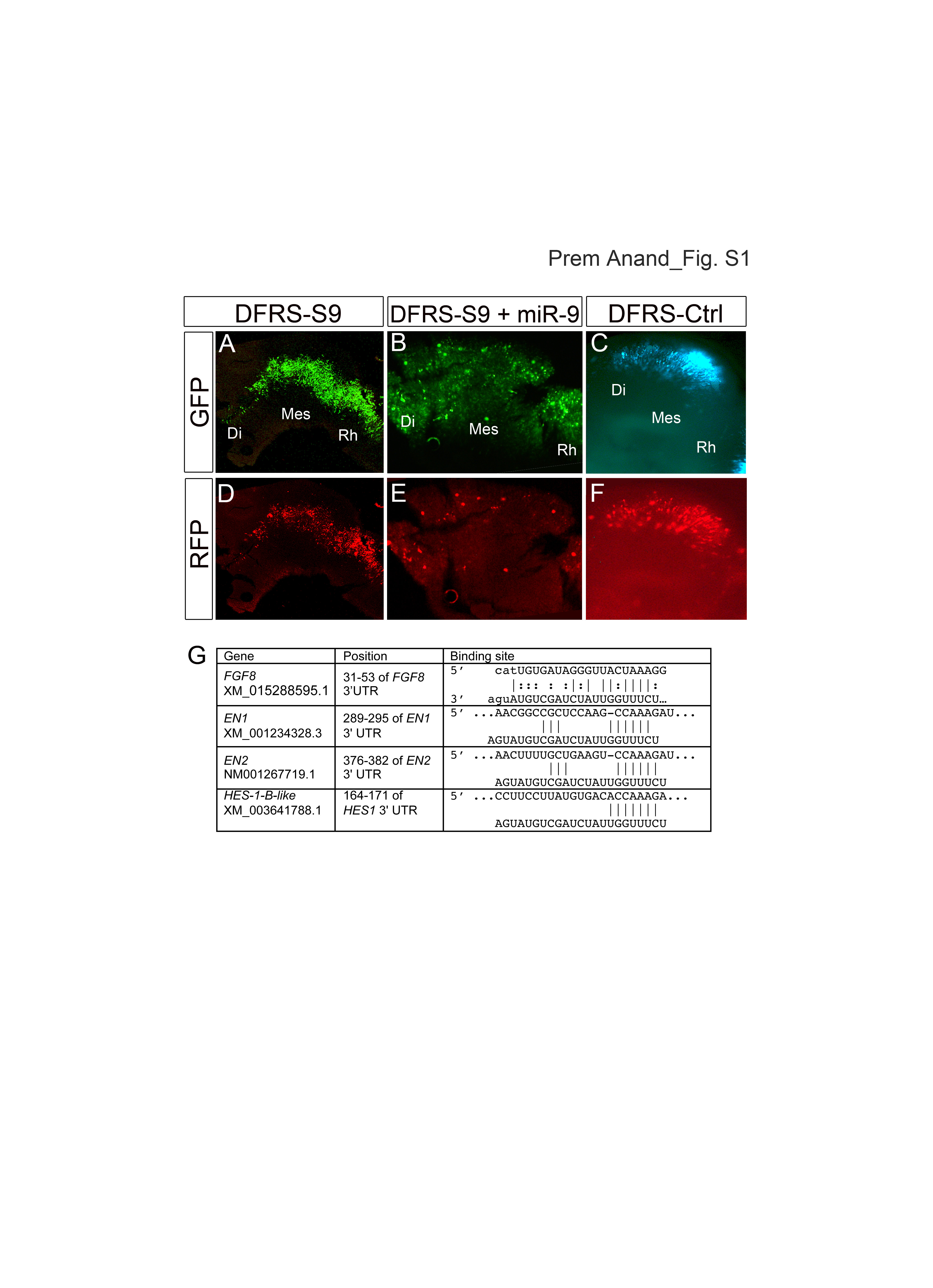

Supplement: Supplementary file 2 — Functional miR-9 overexpression. (A) depicts wildtype cells expressing GFP after transfections with DFRS-S9 sensor plasmid. The red, RFP expressing cells in (D) are those cells of (A) that do not express miR-9. Overexpression of miR-9 together with DFRS-S9 (B,E) showed that almost all cells in midbrain express only GFP (B) and no RFP (E) and therefore miR-9. With the DFRS-control sensor plasmid all transfected cells express GFP (C) and RFP (F). (G) shows the binding sequences for miR-9 in the 3’UTRs of FGF8, EN1, EN2 and HES1-B-like. Abbreviations: Di-diencephalon, Mes-mesencephalon, Rh-rhombencephalon. (PNG 3031 kb) [file 12861_2017_159_MOESM1_ESM.png]

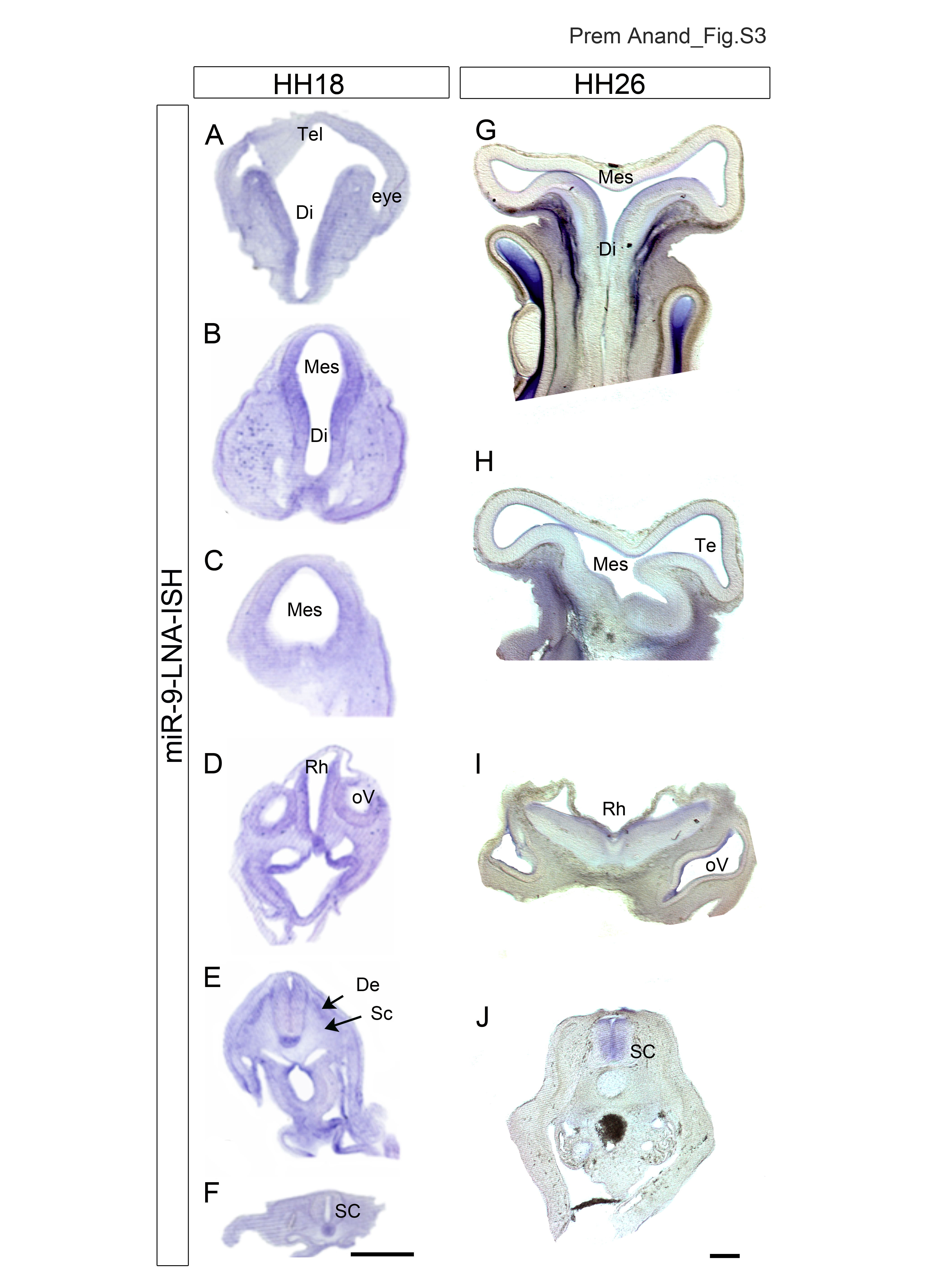

Supplement: Supplementary file 4 — Expression of miR-9 in HH18 and E6 chick brains. Coronal sections through HH18 (A-F) and HH26 (E6; G-J) chick brains. (A-F) are sections on the level of forebrain (A), mesencephalon (B,C) rhombencephalon (D), posterior rhombencephalon (E) and spinal cord (F). (G-J) are sections through di- and mesencephalon (G,H), rhombencephalon (I), and spinal cord (J). Note, miR-9 expression in the ventricular zone of HH26 diencephalon, mesencephalon and rhombencephalon. Abbreviations: De-dermamyotome, Di-diencephalon, Mes-mesencephalon, NC-notochord, oV-otic vesicle, Rh-rhombencephalon, Sc-sclerotome, Te-tectum. Scale bars-100 μm. (PNG 6983 kb) [file 12861_2017_159_MOESM3_ESM.png]

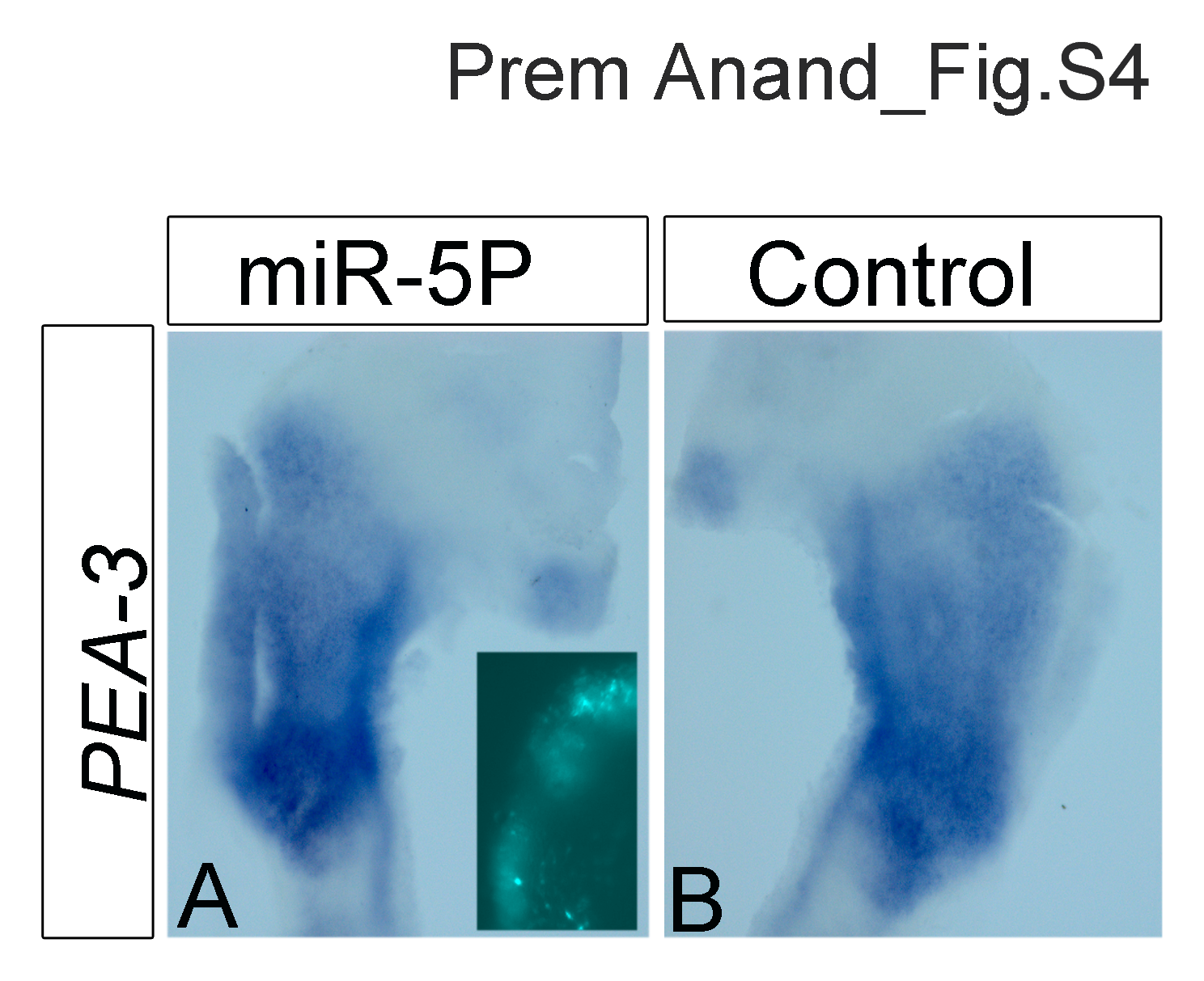

Supplement: Supplementary file 5 — PEA3 expression is unchanged by miR-9 overexpression. Lateral view of the MH level. Left brain half was electroporated with miR-9 duplex (A) at HH10. The untransfected brain half served as control (B). The insert in (B) shows the transfections. PEA3 (blue) was visualised by ISH. (PNG 1230 kb) [file 12861_2017_159_MOESM4_ESM.png]

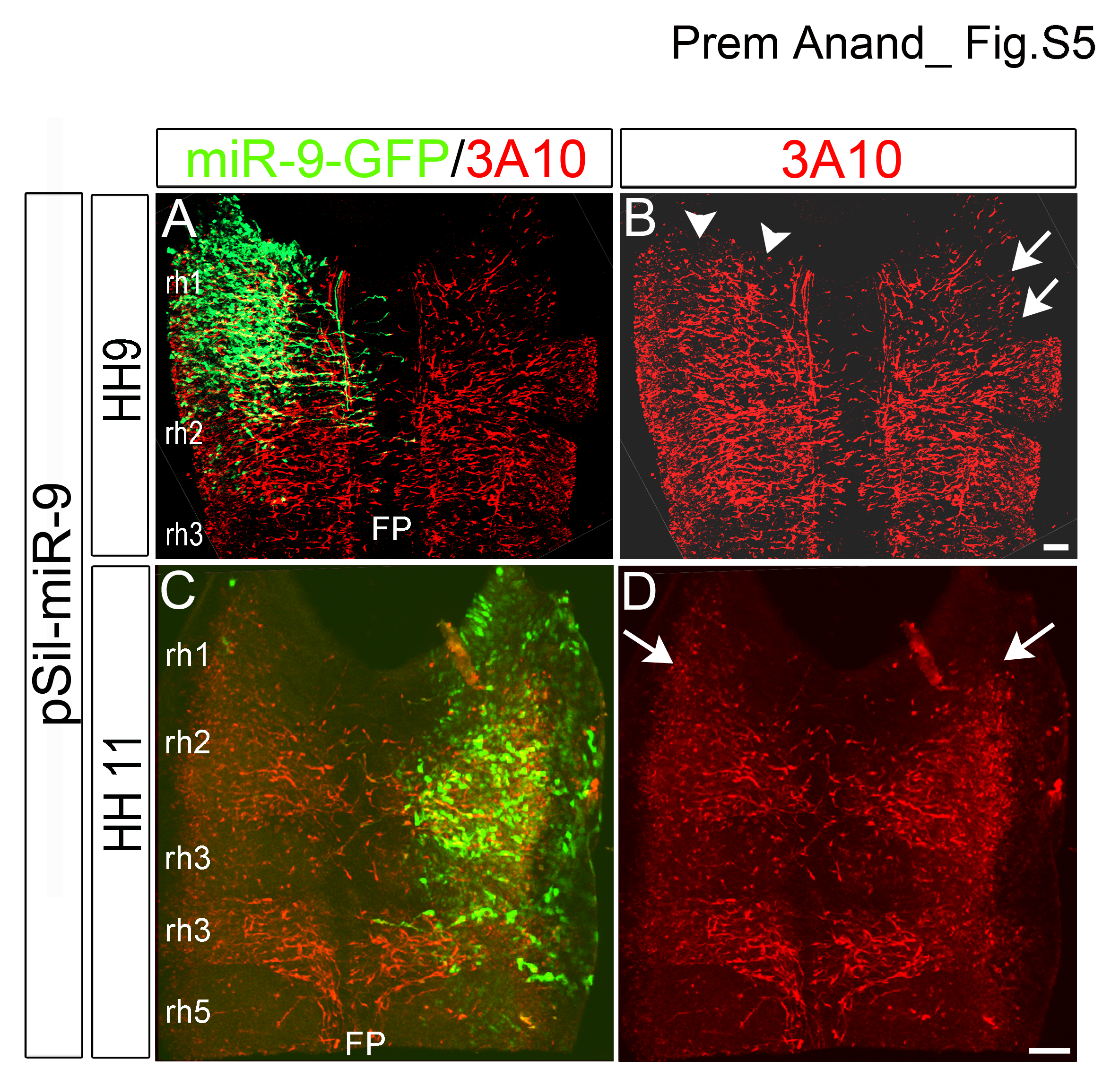

Supplement: Supplementary file 6 — Overexpression of miR-9 in anterior hindbrain promotes neurogenesis. MiR-9 (pSil-miR-9) was ectopically expressed in anterior hindbrain at HH9 (A,B) and HH11 (C,D) in left (A,B) or right (C,D) brain half. The other brain half was used as control. The white arrowheads in (B) point to ‘ectopic’ neurones in rhombomere 1, which are not present in right rhombomere 1 (arrows). Overexpression of miR-9 at later stages (C; HH11) did not result in early neurogenesis in anterior hindbrain (white arrowheads, D). Abbreviations: Rh-rhombomere, FP-floor plate. (PNG 3150 kb) [file 12861_2017_159_MOESM5_ESM.png]

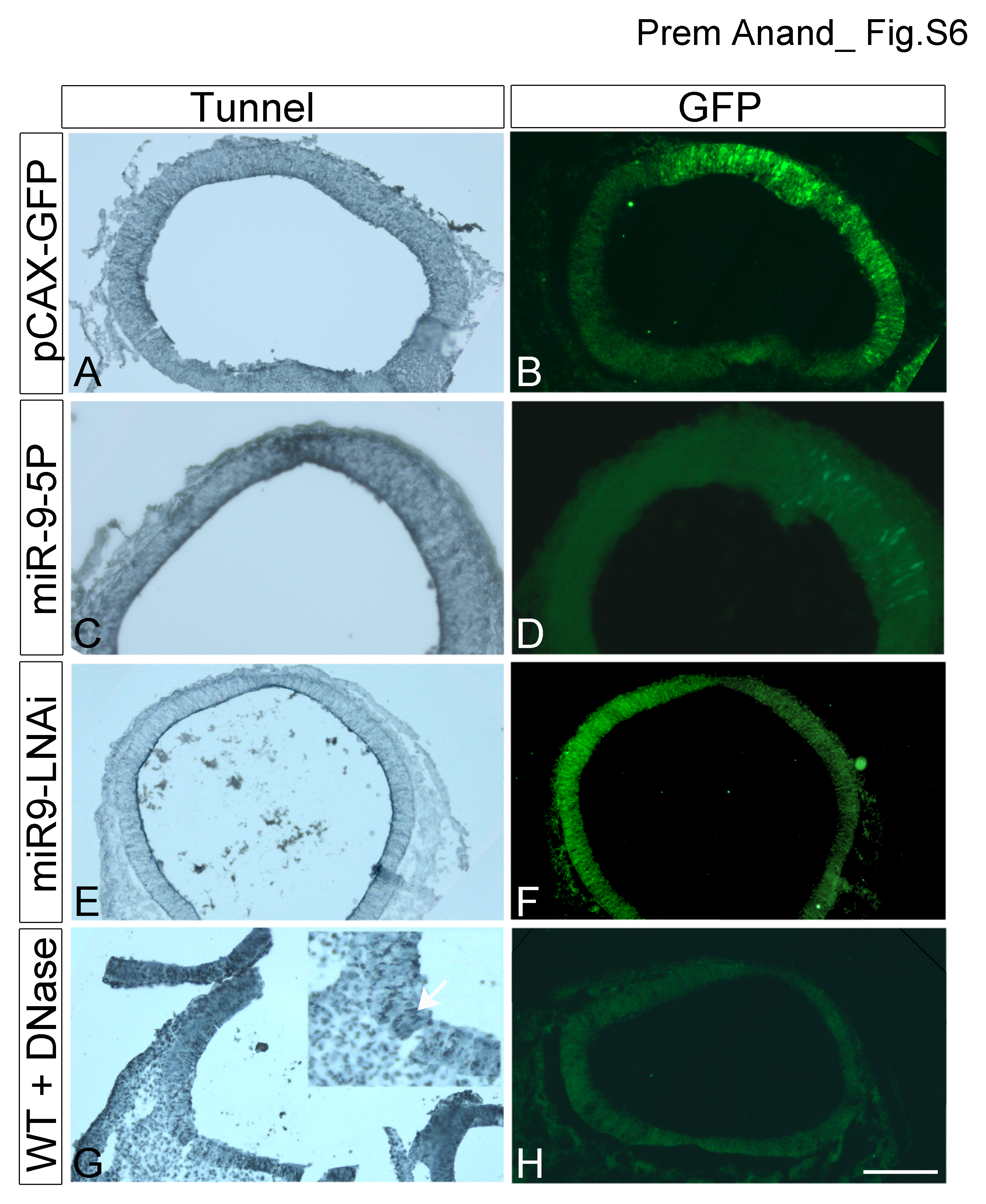

Supplement: Supplementary file 7 — MiR-9 does not raise apoptosis. Section of HH17 midbrains overexpressing pCAX-EGFP (A,B), miR-9-5p (C,D) or miR-9 LNAi (E,F). (G,H) are wild type sections treated with DNase (G) to evoke a positive reaction of Tunnel staining in cells. The arrow in the magnified insert in (G) shows the black Tunnels stained cell nuclei after DNase treatment. No Tunnel staining was observed in the electroporated and non-electroporated midbrain halves (A-F). Scale bare: 200μm. (PNG 7740 kb) [file 12861_2017_159_MOESM6_ESM.png]

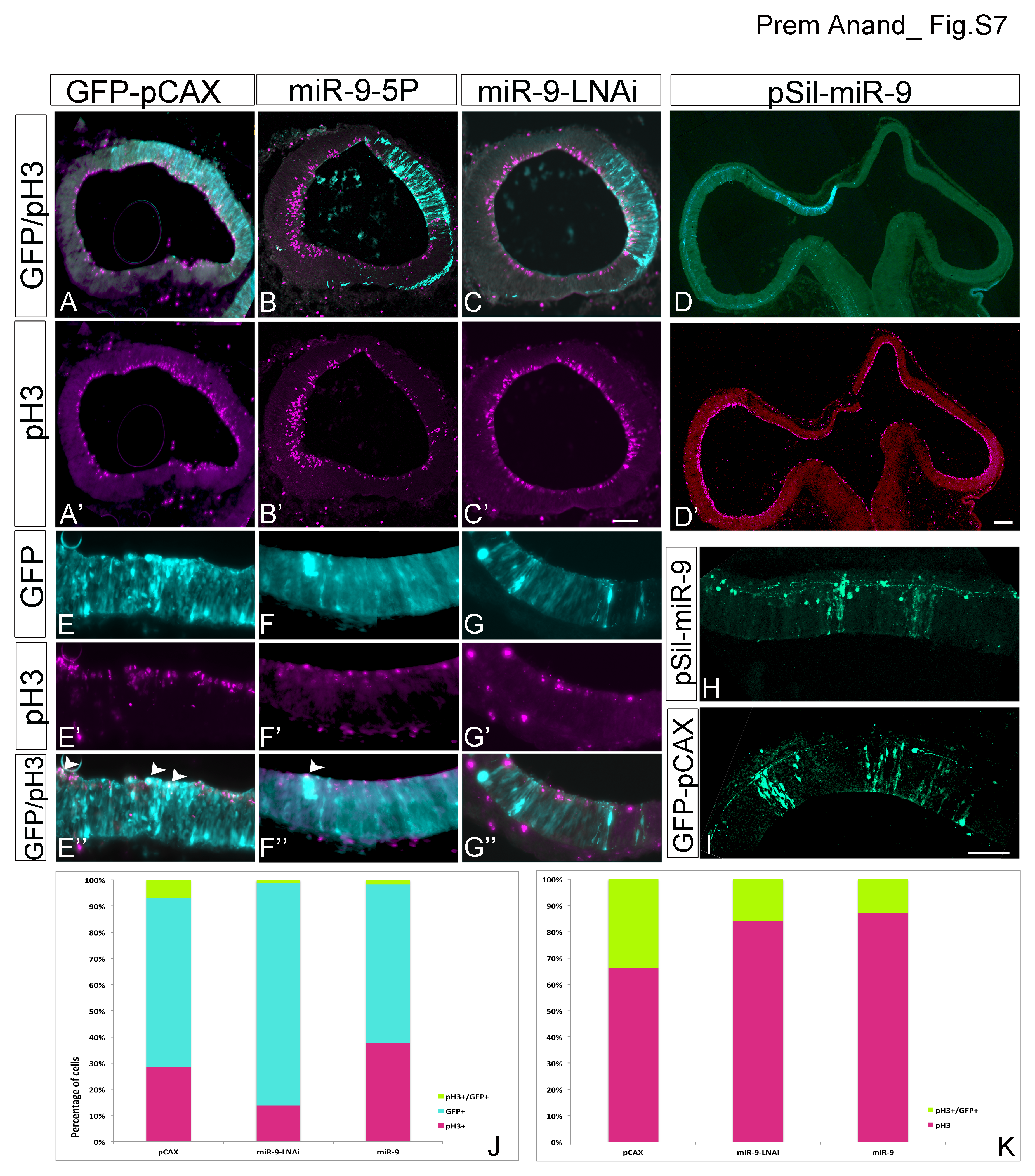

Supplement: Supplementary file 8 — MiR-9 misexpression and mitotic cells. Section of HH17 midbrains overexpressing pCAX-EGFP (A,A’,E,E’,E”’,I), miR-9-5p (B,B’F,F′,F″) or miR-9 LNAi (C,C’,G,G’,G”). Sections of HH26 midbrain transfected with pCAX-EGFP (I) and pSil-miR-9 (D,D’,H). Sections were immunostained for EGFP (green) and pH 3 (red). (A-C, E”-G”) show the overlays of GFP and pH 3 expressing cells. (E-G”) are magnifications of (A,B,C), respectively and (H) is a magnification of (D). Several EFGP+/pH 3+ cells are indicated by arrowheads in (E”) and (F″). The magnifications in (H) shows more GFP+ cells in the mantle zone than the control (I). (J,K) are percentage graphs displaying the percentage average of GFP+, pH 3+ and GFP+/pH 3 cells after different treatments of midbrain. (K) shows the percentage of pH 3+/GFP+ cells of pH 3+ cells. Midbrain cells expressing only GFP are almost two thirds more likely to express pH 3. Scale bars in (C’, I): 100 μm; scale bare in (D’): 200 μm. (PNG 7500 kb) [file 12861_2017_159_MOESM7_ESM.png]
