# Supplementary material for: Expression and function of microRNA-9 in the mid-hindbrain area of embryonic chick
Source: BMC Dev Biol. 2018 Feb 22;18:3. doi: 10.1186/s12861-017-0159-8 (PMC5824543; doi:10.1186/s12861-017-0159-8)

Prem Anand_Fig. S2

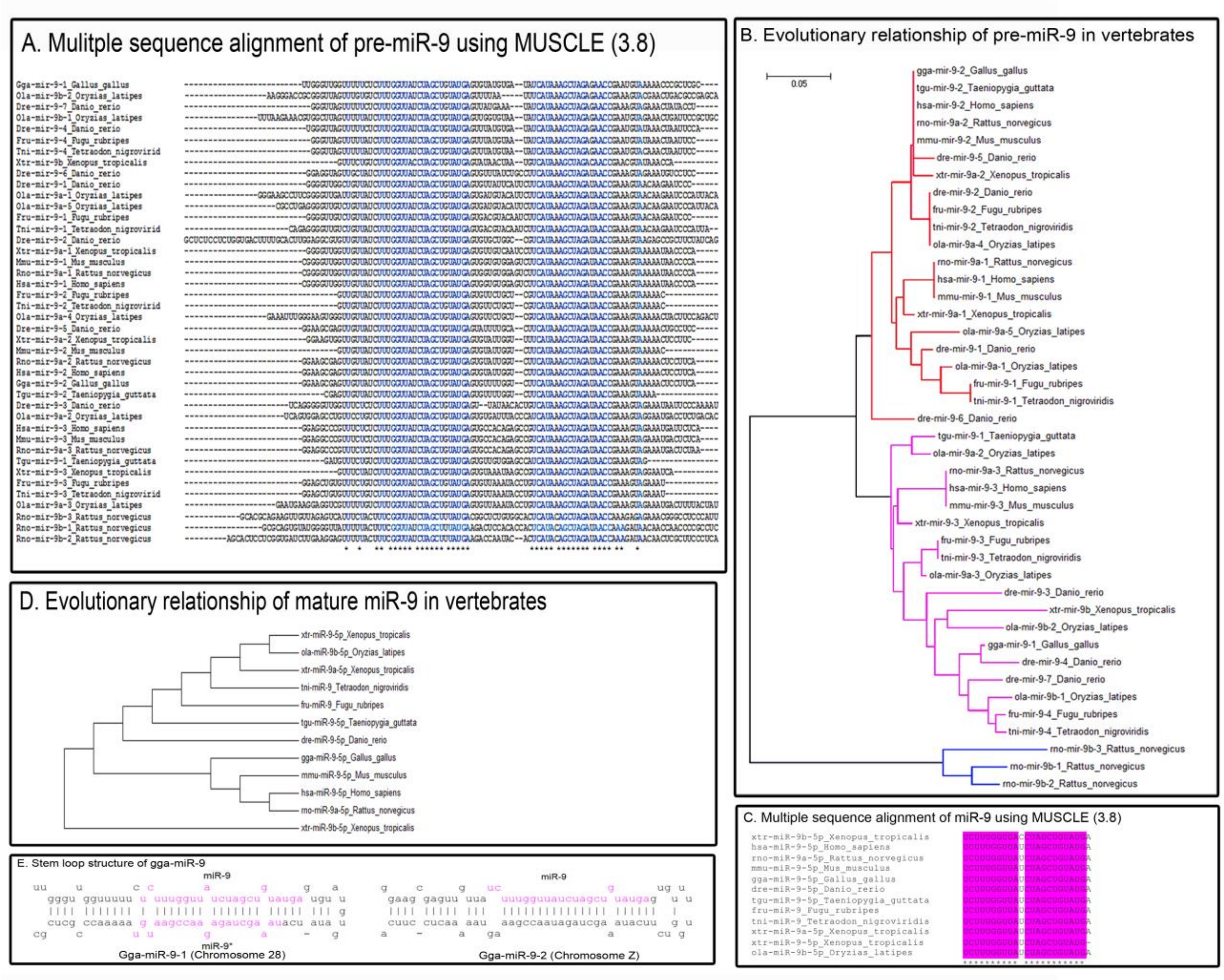

Supplement: Supplementary file 3 — A. CLUSTAL multiple sequence alignment of pre-miR-9 sequence by MUSCLE (3.8) shows that throughout vertebrate species miR-9 is conserved in the stem region of the hairpin (indicated by blue letters and asterix marks). B. Evolutionary relationship of pre-miR-9 family. The Neighbour-Joining phylogram shows the phylogenetic relationships of pre-miR-9 variants among vertebrates. The evolutionary distances were computed using the Maximum Composite Likelihood method and the branch lengths are proportional to the number of base substitutions per site. The scale bar indicates substitution rate of nucleotides per site. Evolutionary analysis was performed in MEGA6. The colour denotes the Clades of miR-9 family in vertebrates (Clade I: red, Clade II: Rose and Clade III: Blue). Clade I has two subclades, namely miR-9-1 and miR-9-2. Clade II has miR-9-3 and miR-9-4. Clade III consists of miR-9 from Rattus norvegicus. C. CLUSTAL multiple sequence alignment of mature miR-9 sequence by MUSCLE (3.8). The conserved sequences are indictaed in pink and with asterix marks. D. The cladogram shows the evolutionary relationship of mature miR-9 among different vertebrates. Branch lengths are not proportional to the sequence divergence. E. The stem loop structure of Gallus gallus miR-9 variants. The gga-miR-9-1 gives raise to functional mature miR-9 (miR-9-5p) and miR-9* (miR-9-3p), while gga-miR-9-2 only produce miR-9 (shown in colour). The mature miR-9 obtained from both gga-miR-9-1 and gga-miR-9-2 has the same sequences and evolutionarily conserved. (PDF 369 kb) [file 12861_2017_159_MOESM2_ESM.pdf]
